# Distribution of and associated factors for dengue burden in the state of Odisha, India during 2010–2016

**DOI:** 10.1186/s40249-019-0541-9

**Published:** 2019-05-06

**Authors:** Subhashisa Swain, Minakshi Bhatt, Sanghamitra Pati, Ricardo J. Soares Magalhaes

**Affiliations:** 1 Indian Institute of Public Health-Bhubaneswar, Public Health Foundation of India, Bhubaneswar, Odisha India; 2 Regional Medical Research Center, Indian Council of Medical Research, Bhubaneswar, Odisha India; 3 School of Medicine, University of Nottingham, Nottingham, UK; 4 UQ Spatial Epidemiology Laboratory, School of Veterinary Science, The University of Queensland, Gatton, QLD 4343 Australia; 5 UQ Child Health Research Centre, Children’s Health and Environment Program, The University of Queensland, South Brisbane, QLD 4101 Australia

**Keywords:** Disability adjusted life year, Dengue, Burden, Distribution, India

## Abstract

This study is aimed to estimate the epidemiological burden of dengue in Odisha, India using the disability adjusted life year (DALY) methods and to explore the associated factors in the year 2010–2016. During the period of 2010–2016, 27 772 cases (68.4% male) were reported in the state. Mean age (years) of male and female was 31.63 and 33.82, respectively. Mean district wise disability adjusted life years (DALY) per 100 000 people was higher in the year 2016 (0.45) and mean DALY lost per person was highest in the year 2015 (34.90 years). Adjusted regression model indicates, every unit increase in humidity and population density increases DALY by 1.05 and 1.02 units respectively. Whereas, unit change in sex ratio (females per 1000 males) and forest coverage increases the DALY by 0.98 units. Our results indicate geographical variation of DALY in Odisha, which is associated with population density, humidity and forest cover. Discrepancies identified between standard incidence and DALY maps suggests, latter can be used to present disease burden more effectively. More prevalence among young males suggests the need of strengthening the targeted prevention and control measures.

**Electronic supplementary material:**

The online version of this article (10.1186/s40249-019-0541-9) contains supplementary material, which is available to authorized users.

## Multilingual abstracts

Please see Additional file 1 for translations of the abstract into the five official working languages of the United Nations.

## Background

Clinical dengue inflicts significant health, economic, and social burden on the people. The World Health Organization (WHO) estimates 390 million dengue infections annually and approximately 3.9 billion people live in dengue endemic countries [1]. Nearly 75% of the global burden of dengue is located in the South-East and Western Pacific Regions [2]. There has been 30-fold increase in dengue cases over the past 50 years in 119 countries [3]. In India, dengue notification reflects seasonal patterns, which over the years have increased in frequency and geographical extent [4, 5]. For example, the number of dengue notifications increased from15 535 cases in the year 2009 to 40 425 cases in the year 2014 [6]. Whereas, the maximum number (75 808) was reported in the year 2013 and the maximum number of deaths (242) was reported in the year 2012 [6]. This increase is particularly noteworthy because available dengue notification data is largely derived from hospitalized cases of dengue hemorrhagic fever (DHF) and dengue shock syndrome (DSS) [7].

Despite various efforts to control or prevent the transmission of dengue virus in India, it is still a threat to the public health [8]. In earlier years, dengue was confined to some urban areas of the country, however, currently it is being reported from all the states. Until the year 2008, Odisha (eastern Indian state) had no reported cases of dengue [9], and the first outbreak was reported in the year 2010. According to the surveillance data, the state now contributes nearly 10–15% total dengue cases of the country. These cases are scattered in distribution and uneven with circulation of four serotypes of the virus. In the state, increased numbers of cases among children and adults has been reported [10–12]. Considering the age-shift in disease and geographical distribution of the dengue, quantification of disease burden and identification of associated risk factors would generate much needed evidence for informed decisions regarding resource allocation and the design of intervention trials.

The dengue scientific working group recommends estimation of dengue burden as priority in the global dengue research agenda [13]. Despite the global expansion and clinical importance of dengue, few studies have assessed the burden of this disease in India, let alone for the high endemcity districts of Odisha [14]. In epidemiology, incidence and prevalence are commonly used indicator for explaining the burden of the disease. However, it is essential to have a comprehensive method for estimating the burden considering both cases and death together along with population structure. Murray and Lopez [15] brought the concept of calculating the burden of disease through disability adjusted life years (DALY) [16]. DALY measures the burden of the condition by measuring the quality of life reduced by disability and lives lost due to premature death. Substantial consequences do exist on use of DALY as an indicator, because of the consideration of overall aspect of the disease and standardized methods and considering the disability weights for the calculation of population-based burden [16]. Understanding the factors associated with the burden also important for better preparedness.

Mostly, dengue outbreaks are linked to number of biotic and abiotic factors including climate variability indicators such as rainfall, temperature and humidity [17, 18], population growth, urbanization, lack of sanitation, increased human travel, ineffective mosquito control, and better surveillance system [19–21]. Other factors include health system readiness, capacity of healthcare systems, effectiveness of vector control systems, predominant circulating dengue serotypes, herd immunity, and social behaviour of the population [22, 23]. However, little is known about the associated factors in the state and the country.

This study aims to estimate the dengue burden within the state of Odisha using both epidemiological indicators and DALY, understand their geographical distribution and to explore the associated factors (climatic, demographic and geographical) at the region level.

## Materials and methods

### Study site

Our study is from the Odisha state of the India, located between the parallels of 17.49′N and 22.34′N latitudes and meridians of 81.27′E and 87.29′E longitudes, having 480 km of coastline along the Bay of Bengal on the East. The state has three broad distinct morphological features: coastal plains, southern mountainous and plateau, western rolling uplands. According to the 2011 census of India, the total population of Odisha was 41 947 358 of which 49.46% are female and the population density of the state was 269 per square kilometre [24]. In a year, the maximum temperature ranges between 35 and 40 °C and the lowest temperatures are usually between 12 and 14 °C. The average rainfall is 150 cm, experienced as the result of south west monsoon during July–September [25].

### Dengue data

Data on dengue cases and deaths for all districts of the state was obtained from the National Vector Borne Disease Control unit, Odisha, India for the period of 1st January 2010 to 31st December 2016. The cell collects epidemiological data from both public and private health facilities in the state. Surveillance data for dengue are collected in two ways. Firstly, any suspected cases of dengue are identified by the community health workers or the physicians based on the given case definition (an acute febrile illness of 2–7 days duration with two or more of the listed manifestations: headache, retro-orbital pain, myalgia, arthralgia, rash, haemorrhagic manifestations, leukopenia). Later, confirmatory diagnosis is done by Non-ELISA based NS1 antigen/IgM positive laboratory test [26]. All the patients reported to either public or private hospitals are screened through the serum test and the data are routinely reported to the disease control cell. For our study, only laboratory confirmed cases were included. The anonymized database used in the study had information on types of the hospital/ clinic/laboratory, date of diagnosis, age, sex, date of discharge, outcome of the disease, address of the patient, and method of diagnosis. We did not have complete line listings and information for all the study years. Details of the information available in each year is given supplementary file. So, we used different year data for different estimation purposes. For example, as information for the year 2011 and 2012 was incomplete, for the estimation of DALY, data of the years 2013–2016 was used. However, the data of the years 2010–2016 was used for mapping dengue incidence and deaths. Cases, which had incomplete information (*n* = 37) on age or sex, were contacted by the investigators through available contact numbers and information was updated and considered in the analysis.

### District level information

In dengue transmission, population factors are emerging along with other climatic and environmental factors [22]. For this purpose, we collected district level information on the population, sex ratio, literacy rate and population density form 2011 India census. As the population of districts in each year was not available during the period between 2012 and 2015, it was estimated using the annual growth rate available at census data. Age and gender specific population for all the districts was estimated using annual growth rate data with reference to the state census data of 2011. Then, age was categorized based on 5 years age groups, as per the WHO template. We collected information on number of industries and urban areas in a district from the website of the department of urban and industry, Government of Odisha available for the year 2014.

### Weather data

Evidence suggests a strong relationship between weather change and dengue. The information on annual average temperature, rainfall and humidity for the study districts for the study years 2013–2016 was collected from Indian Meteorological Department (IMD) [18]. IMD measures the climatic parameters from the 23 weather stations located in the state. The information on forest coverage for the year 2009, 2011 and 2013 was derived from the available data in the website portal [27]. The forest coverage was measured in percentage of the districts are covered with forest, measured using satellite imaging techniques. We calculated temperature humidity index using the formula described by Perry et al. [28]. Studies reported that, temperature and humidity are highly correlated with distribution of the dengue [29], however, there is lack of evidence on using climatic index rather than standalone climatic factors.

### Dengue DALY estimation

We used the template available at WHO website for estimating region specific DALY [30]. The district specific DALY was estimated for both males and females using the appropriate disability weight recommended by WHO described in supplementary file [31]. The region specific choropleth maps of incidence (per 10 000 population) and DALY (per 100 000 population) were produced using the software Quantum GIS (version 3.2.0, Opensource, Switzerland) for the years 2011 to 2016 [32]. The shape file of the state was obtained from Odisha Space Application Center (ORSAC) [33].

### Statistical analysis of factors associated with DALY for dengue

We used district as the geographical unit and every year as the temporal unit for the analysis. The dengue cases in each district were reported in the absolute numbers and we calculated DALY for each district for each of the study year. The aim was to have a standardized estimate for each district considering the total population and deaths. The district level DALY per 10 000 population was used as an outcome in regression model to identify possible factors. We tested for collinearity between the variables using the variance inflation factor in post model estimation. The generalized linear negative binomial regression model with ‘log-link’ function was used to estimate the indicators for district wise DALY using different covariates. This model accounts the non-normal distribution of residuals and reported higher number of zeros DALYs in the data. Backward step wise regression was used to select variables for the final model. At each step, the variable having highest insignificant *P* value (*P* > 0.05) was removed from the model, till we reached at a model with all the variables having significant *P* value. The models were tested using likelihood ratio test to decide best-fit one. The adjusted estimate was obtained considering all variables together. The findings are reported as incidence rate ratios with 95% *CI* at the significance level of < 0.05. As year was not significantly associated with deaths or cases, we used year as fixed effect in the model; all analysis was conducted using the software STATA (V.12, STATA corp, Texas, USA).

## Results

### Dataset for analysis

**Fig. 1:**
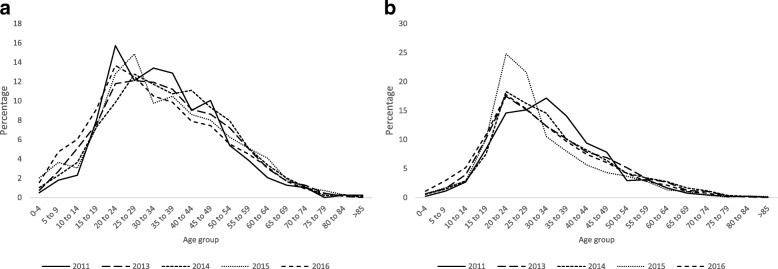
**a** Distribution of cases across age in different years in males. **b** Distribution of cases across age in different years in females

**Fig. 2:**
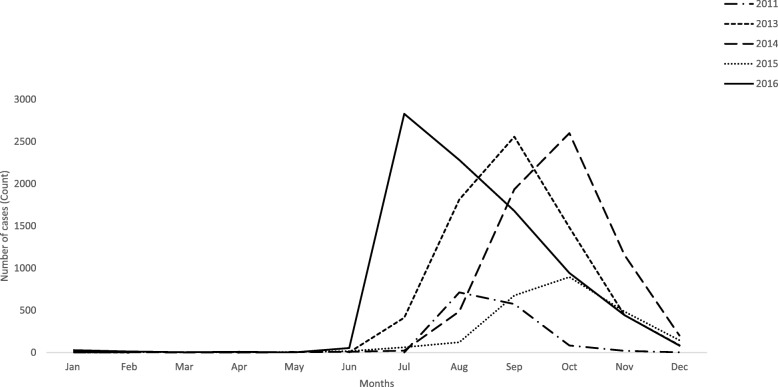
Seasonality of cases per 10 000 population, month wise across three years

In six years (2010–2016), a total of 27 739 dengue patients (including the patients in 2012) were reported in the state of Odisha. There was variation in number and distribution of cases over the years. (Table 1) Maximum number of cases (*n* = 8304) were observed in the year 2016. Out of total cases, 31.6% were female. Each year, the number of reported male cases were higher than females (Chi square: 111.45, df = 5, *P* < 0.001). The mean age of male and female were 31.63 years (*SD*: 13.88 years) and 33.82 years (*SD*: 15.41 years), respectively and the trend of mean age distribution was statistically significant (Cochrane-Armitage Trend test, *P* < 0.05) (Table 1). Among males, more cases were reported in the age group 20 to 34 years whereas, among female most of the cases are in the age group of 20 to 30 years (Fig. 1 a & b). The mean annual temperature, rainfall and temperature humidity index (THI) were found to be different across the year with significant trend (*P* < 0.05). The month-wise distribution of cases shows an early reporting of index cases in a year (during early monsoon period) in the recent year 2016 compared to previous years (Fig. 2).

**Table 1:** Descriptive statistics of variables between 2011 and 2016

Variables	2011 (*n* = 1718)	2013 (*n* = 6813)	2014 (*n* = 6206)	2015 (*n* = 2424)	2016 (*n* = 8304)	Cochrane Armitage Trend test (*P* value)
Male (%, 95 *CI*)	77.41 (75.43–79.39)	65.61 (64.49–66.74)	68.65 (67.49–69.81)	71.69 (69.90–73.49)	67.64 (66.64–68.65)	Chi square- 111.45, df = 5, (< 0.001)*
Female (%, 95 *CI*)	22.58 (20.60–24.56)	34.38 (33.25–35.50)	31.34 (30.18–32.50)	28.30 (26.50–30.09)	32.35 (31.34–33.35)	
Age in year (Mean, 95% *CI*)	32.89 (32.29–33.50)	32.98 (32.62–33.35)	33.65 (33.29–34.01)	31.00 (30.44–31.55)	31.12 (30.80–31.43)	< 0.001*
Temperature in degree Celsius (Mean, 95% *CI*)	NC	29.07 (28.67–29.47)	30.78 (30.68–30.89)	31.62 (31.54–31.69)	30.03 (29.60–30.46)	3.51 (< 0.001)*
Rainfall in mm (Mean, 95% *CI*)	NC	1609.48 (1505.85–1713.11)	1452.39 (1360.24–1544.53)	1289.45 (1209.60–1369.31)	1433.83 (1316.27–1551.40)	−3.12 (0.002)*
Humidity % (Mean, 95% *CI*)	NC	61.68 (59.88–63.47)	59.69 (59.95–62.42)	55.12 (52.93–57.32)	61.25 (59.44–63.05)	−1.36 (0.173)
THI (Mean, 95% *CI*)	NC	21.55 (21.35–21.74)	22.36 (22.31–22.41)	22.72 (22.68–22.76)	22.01 (21.80–22.22)	3.50 (< 0.001)*
Death in number (count)	33	6	9	2	11	
Regional DALY per 100 000 population (Mean, 95% *CI*)	NC	0.09 (−0.03 to 0.21)	0.15 (− 0.01 to 0.31)	0.45 (− 0.04 to 0.93)	0.15 (− 0.02 to 0.33)	1.05 (0.292)
DALY per person in year (Mean, 95% *CI*)	NC	30.43 (15.38–45.30)	33.52 (29.05–37.69)	34.90 (25.64–43.27)	29.10 (12.32–45.58)	

*significant at *P* value < 0.05; *THI*: Temperature-humidity index; *CI*: Confidence interval;

(Total cases in 2010 was 34; Total cases in 2012 was 2240. Because of unavailability of complete line listing, descriptive statistics could not be estimated for both the years. NC: Climatic data for the year 2011 was not analysed, as this year’s data was not included in the model)

**Fig. 3:**
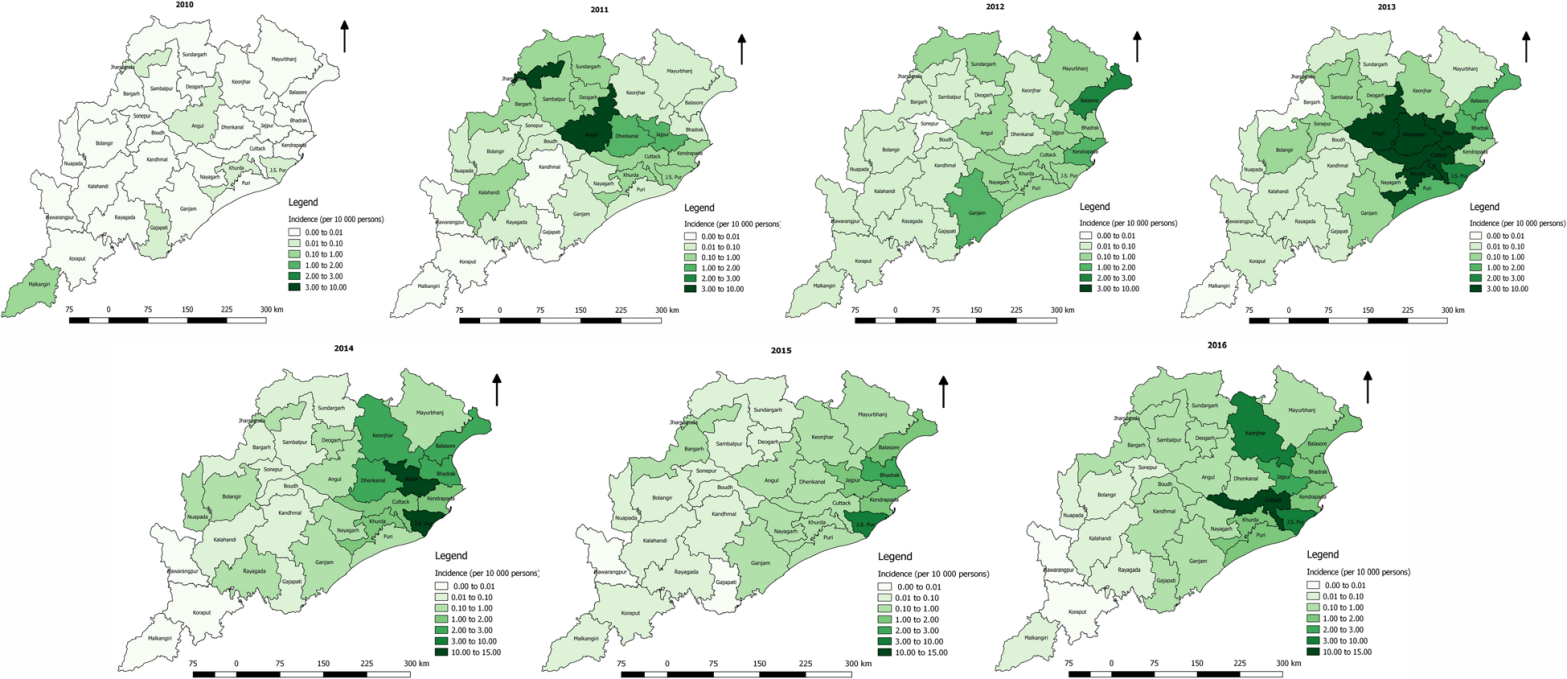
Incidences of dengue cases per 10 000 persons from 2010 to 2016

**Fig. 4:**
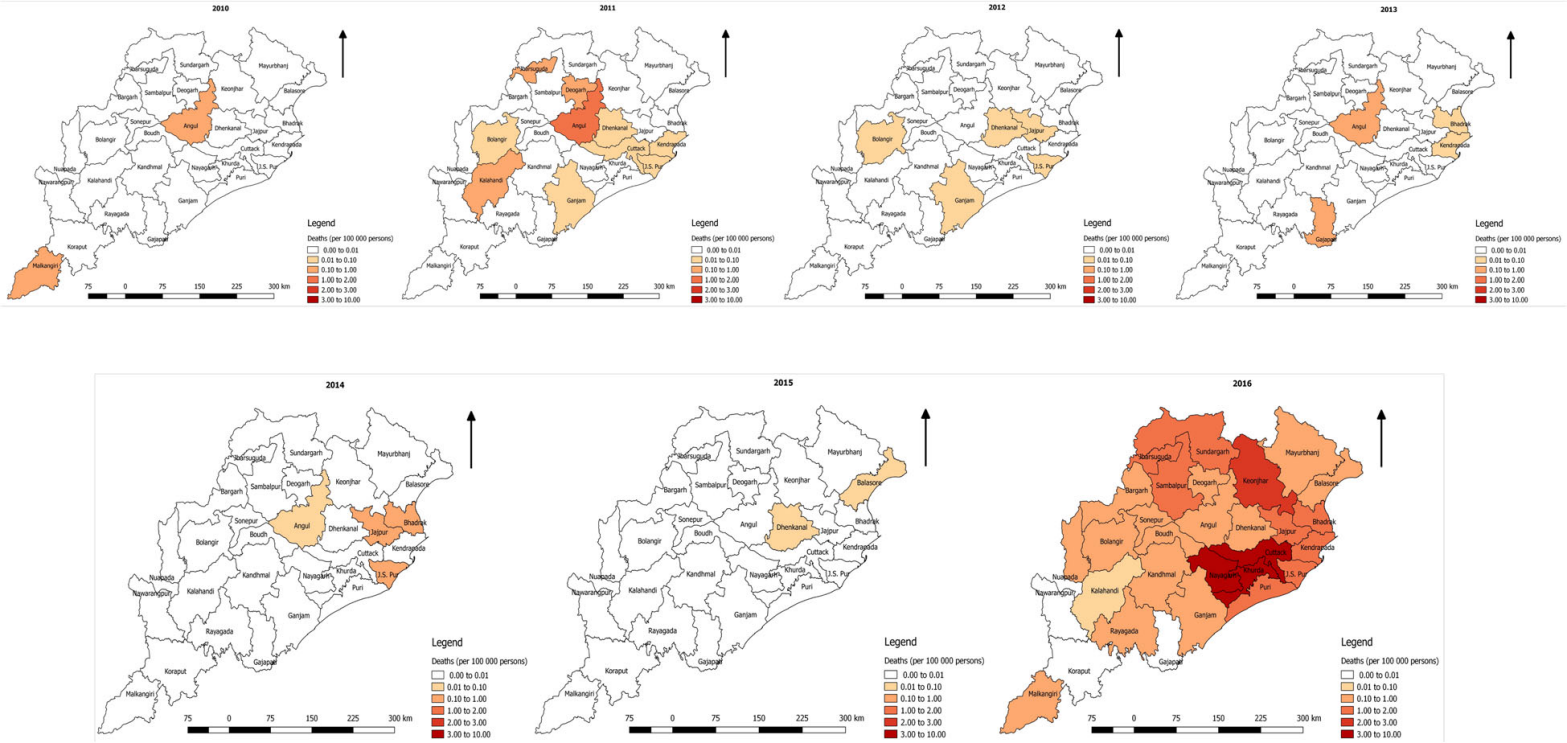
Death due to dengue per 100 000 persons from 2010 to 2016

Eight of 30 districts reported dengue in the year of 2010 which later expanded to 25 districts in the year 2011 and 29 districts in 2012. During the year 2011, only 4 districts (Angul, Cuttack, Jajpur and Jharsuguda) reported more than 2/3 of the total case. In subsequent years, the distribution was geographically wider, but districts namely, Cuttack, Jajpur and Bhadrak reported higher proportion of the total cases. The incidence rate in the year 2010 was less than 1/100000 population, which has gradually increased over the period and was consistently high in coastal districts. The spread of the incidence (per 10 000 population) and deaths (per 100 000 population) has been given in Figs. 3 and 4, respectively. Incidence map shows, in the last three years, the distribution was expanded to all the districts with higher reporting from the coastal districts (Fig. 3). During the study period, deaths reported, has been temporally irregular (Table 1). Reported deaths due to dengue was sporadic in geographic distribution. However, the geographical distribution of deaths across different years has expanded from 8 districts in the year of 2010 to 27 districts in 2016 (Figs. 3 & 4). There has been no constant reporting of deaths from any districts. In the year 2011, ten of the 30 districts reported deaths, which was maximum but was confined to 3–5 districts in subsequent years.

### Temporal and geographical variation in DALY

**Fig. 5:**
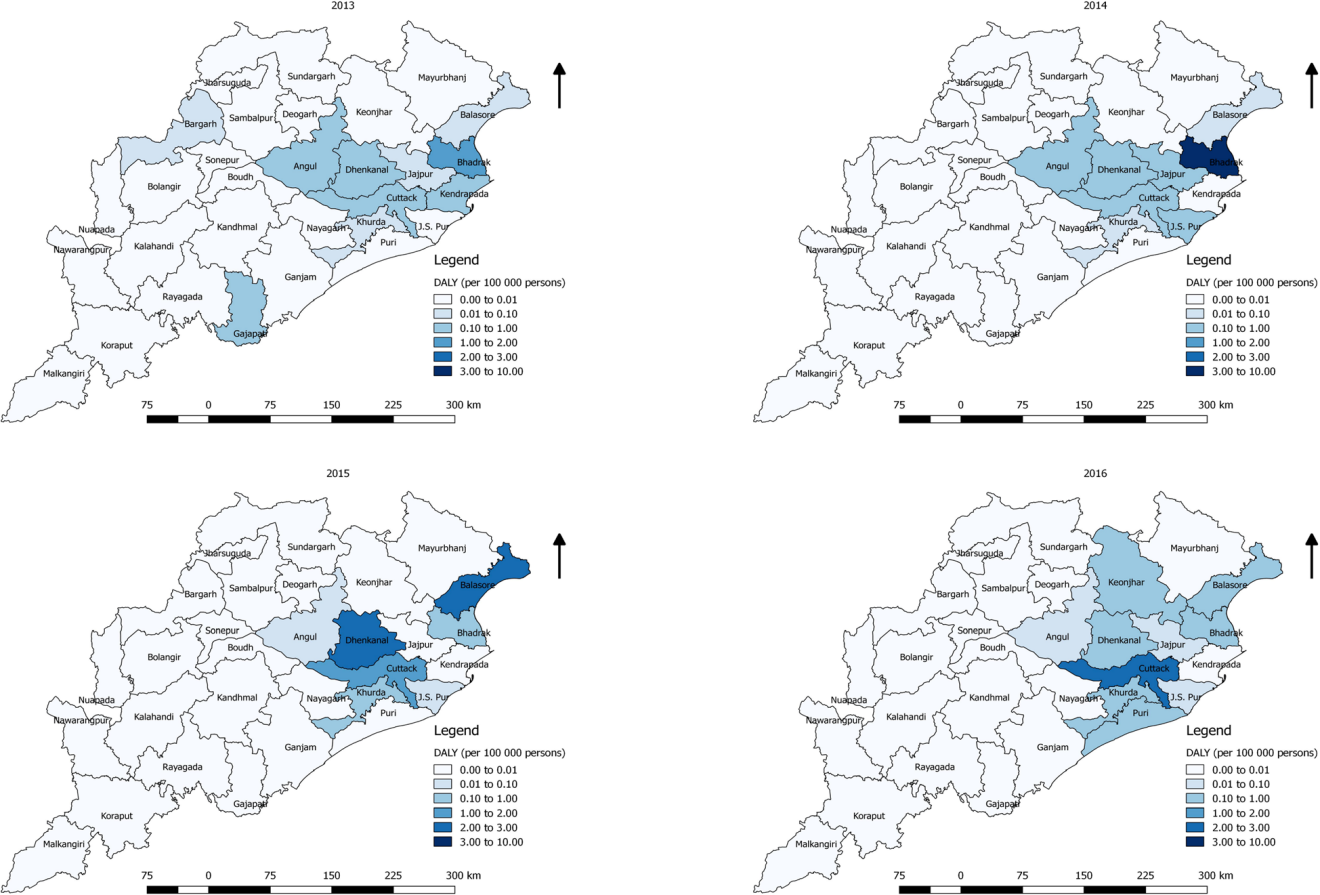
Distribution of burden (regional DALY per 100 000 persons) from 2013 to 2016

The mean district level DALY per 100 000 people was 0.45 in the year 2016 and the mean DALY loss per person was highest in the year 2015 with 34.90 years. The central and coastal districts (Angul, Cuttack, Jajpur, Jagatsinghpur, Bhadrakh and Balasor) had the highest DALY per 100 000 population per year compared to the rest of the districts of Odisha (Fig. 5). In the year 2016, nine out of thirty districts reported DALY to be more than 1/100000 population.

### Factors associated with the variation in DALY

In the final multivariate model, the burden of dengue was found to be significantly associated with sex ratio, population density, forest coverage, average temperature after adjusting for years (Table 2). Every unit increase in humidity and population density, found to be associated with increase in DALY by 1.05 and 1.02 units respectively. Similarly, a unit change in the sex ratio and forest coverage is associated with an increase in the risk of the burden by 0.98 times after adjusting for other variables. We did not find significant association with rainfall in univariate model and with literacy rate and economic status of the district in the multivariate model.

**Table 2:** Unadjusted and adjusted negative binomial model for estimating DALY per 100 000 population

Variables	Unadjusted IRR (95% *CI*)	Final best fit model Adjusted IRR (95% *CI*)
Sex ratio (Number of females per 1000 males)	0.96(0.93–0.98)*	0.98(0.97–0.99)*
Literacy rate (%)	1.09(1.07–1.11)*	
Below poverty line (%)	1.04(1.00–1.08)*	
Population density (per square kilometre)	1.01(1.004–1.02)*	1.02(1.01–1.04)*
Urban areas (%)	0.97(0.89–1.05)	
Number of industries	1.02(1.01–1.03)*	
Forest coverage (%)	0.98(0.97–0.99)*	0.98(0.96–0.99)*
Average yearly temperature (in Celsius)	0.69(0.59–0.81)*	0.71(0.57–0.89)*
Average yearly rainfall (in mm)	1.01(0.99–1.04)	
Average yearly humidity (%)	1.05(1.01–1.08)*	1.05(1.01–1.09)*
Heat index^a^	0.48(0.35–0.67)*	
Year		
2013	Reference	Reference
2014	1.03(0.61–1.74)	2.25(1.11–4.55)*
2015	0.38(0.22–0.66)*	1.72(0.75–3.95)
2016	1.04(0.62–1.76)	2.02(1.04–3.93)*

*Significant at *P* value < 0.05; *IRR* Incident rate ratio; *CI*: Confidence interval

^a^Heat index was dropped from final model because of collinearity

## Discussion

In Odisha, dengue is gradually spreading all over the state. This paper describes the distribution of dengue cases in the state during past 7 years and its’ burden for the past 3 years. We also estimated the factors associated with the burden at district level. To our knowledge, this is first ever study from India to explain the sub-regional variation in the burden of the dengue and explored associated factors with regional-burden rather than only incidence. Our key findings are, 1) there is rise in cases in early months of the year compared to previous years, 2) significant spatiotemporal clustering of dengue cases in coastal districts, 3) males (in the age group 20–40 years) are predominantly affected compared to females, 4) gender disparity, population density, forest coverage, average temperature and humidity are strongly associated with the district level burden of dengue in Odisha.

Traditionally, dengue notifications in India are post-monsoon (month of August) and the reported cases attain peak in during the month of September [34]. The incident is because of abundant stagnant water sources for mosquito breeding following heavy rainfall [34–37]. This study in Odisha extends existing knowledge that, even though cases consistently peak in September, more cases during the month of June/July is recent phenomenon. This could be due to the increased container breeding sites during summer e.g. by the wider use of air conditioners. However, we do not have any official statistics to support our assumption. In addition, in recent years, Odisha experiences unexpected heavy rain during summer [38] which leads to accumulation of waters in containers especially in tiers, coconut shells and plastic containers. So, these earlier temporal trends could be attributed to recent extreme weather events. This can trigger the public health department for executing preparedness and preventive activities which are usually implemented during rainy seasons. Along with it, these extreme weathers create more favourable humid atmosphere for mosquito breeding. Thus, the impact of weather change on dengue incidence needs further investigation in the state.

In the state, young adults were mostly affected with dengue, contrast to higher reporting in children and women, elsewhere [39]. This finding is similar to the results from a hospital based study from the Northern and the Eastern India [11, 34]. Circulation of dengue virus serotypes helps in developing herd immunity among adults [40]. However, circulation of different serotypes in different years causes outbreaks in different areas in the state and compromises the herd immunity [10]. So, may be, the herd immunity among adults makes the younger people more vulnerable for the infection. This age shift of the disease precipitates huge economic burden through substantial losses in productivity. We found males were more affected than females contributing nearly 2/3 of total cases. This could be because of the nature of occupation and travel exposure, as males are more likely to travel, and work compared to females. This finding indirectly indicates the importance of working place and travel on dengue incidence, which needs further exploration.

Dengue reporting in the state have increased in last 5 years, mostly in eastern districts of the state. Population density, socio-economic, climatic and the physical environment; characteristic to eastern coastal districts could be the driving factors. Population density is an important risk factors of infectious disease outbreak [41]. Dengue fever incidence is attributed to the rapid urbanization and industrialisation without proper planning towards the increasing number of cases [42]. A study from western India reported human movement as an intrinsic factor for outbreak of dengue [21]. According to the census 2011, coastal districts of the state are densely populated compared to rest of the state [24], which could be because of the better economy and larger urbanisation. Furthermore, better transport facilities with other cities of the country and state, employment opportunities, and rising economy attracts people from different corners of the country to these coastal areas [43]. Additionally, Odisha experiences huge population movement because of its seasonal agricultural practices, rapidly growing industrial job market and the presence of numerous religious and tourist sites, which predisposes the risk of disease importation. [44, 45] The recent major outbreaks in urbanized and urban areas support the evidence.

Climatically, the eastern coastal districts experience a humid weather during summer and rainy seasons. Literature supports the relationship of climatic factors with dengue outbreaks [18, 19] and amongst all, rainfall, humidity and temperature are closely linked with increasing the vector density either through influencing the reproduction rates or vector movement. Our finding of the higher DALY associated with humidity could be because of enhancement of mosquito breeding [18, 19]. The estimated lower risk of dengue burden with increasing temperature could be related to the variation in daily temperature. Average temperature used in the analysis takes the maximum and minimum temperature in to account during estimation. This indirectly explains the temperature variations across the year. Higher the average temperature indicates lower is the diurnal variation. Recent findings suggest variation in diurnal temperature in a day is closely associated with dengue incidence. In the coastal districts of the state, DTR becomes narrow in soon after the summer (June–August) creating favourable condition for dengue transmission [46]. The protective association with forest coverage indicates, districts having less forests are associated with burden of dengue. Lower forest coverage is likely to be linked to a larger share of urbanised areas which creates more anthropogenic environmental conditions for mosquito breeding and dengue transmission [47]. Detail study on the day wise climatic parameters and burden of dengue with inclusion of population dynamics and urbanization factors will provide a robust prediction model.

The WHO recommended method used for estimating the region wise burden considers number of cases and death and population structure in a region for calculating a comprehensive burden estimate. We emphasize the use of DALYs instead of simple incidence or death for better understanding of burden of the disease. Mostly, geographic distribution of burden estimates has been limited to incidence and prevalence rates. Proposed method of estimating burden per region considers disease severity, death and duration, providing robust estimates. Discrepancies identified between standard incidence map and DALY maps reflects mapping the burden of the disease can be used to more efficiently identify at-risk districts for health preparedness to save lives and economic loss [48]. Increased risk association with dengue burden was seen in the year 2014 and 2016 compared to 2013 is because of higher mortality reported in these years compared to 2015. To the best of our knowledge, this is the first study that attempted to quantify the spatiotemporal heterogeneity in the burden of dengue using time-series historical notification data in India.

The findings of our study need to be interpreted considering some limitations. Firstly, the underreporting of cases, especially from private health institutions can be a problem. However, that should not be a major concern for the study state, because, according to recent survey, nearly 80% of the population in the state visit public facilities [49]. Secondly, the lack of micro-level, climatic data, and social, cultural, behavioural or comorbid characteristics of the individual patients have limited the scope of our analysis. Thirdly, the absence of the information on population dynamics, migration and health services factors could be other limitations in our analysis indicating the need of a large community-based study. Finally, the age/sex differentials and the geographical disparities in deaths and incident cases could be explained by dengue serotype variation but unfortunately this information is not available from the routine surveillance data. We also, acknowledge the use of yearly data, might have introduced less variability which requires careful interpretation of our regression findings.

## Conclusions

Instead of only incidence, DALY burden of the dengue can be used to identify at-risk districts for health preparedness to save lives and economic loss. The clustering of cases in the eastern districts and more prevalence among young males suggests the need of strengthening the prevention and control measures. Early reporting of cases in a year can be used in future prediction modelling. Beyond climatic factors, population density is associated with the burden, which can be considered in prevention planning.

## Additional file

Additional file 1: Multilingual abstracts in the five official working languages of the United Nations. (PDF 406 kb)

## Abbreviations

DALY: Disability adjusted life years
DHF: Dengue haemorrhagic fever
DSS: Dengue shock syndrome
DTR: Diurnal temperature range
ELISA: Enzyme linked immunosorbent assay
GIS: Geo information system
IMD: Indian Meteorological Department
ORSAC: Orissa Space Application Centre
*SD*: Standard Deviation
THI: Thermo-hygroscopic index
WHO: World Health Organisation
