# Supplementary material for: Distribution of and associated factors for dengue burden in the state of Odisha, India during 2010–2016
Source: Infect Dis Poverty. 2019 May 6;8:31. doi: 10.1186/s40249-019-0541-9 (PMC6501402; doi:10.1186/s40249-019-0541-9)
Supplement: Supplementary file 1 — Multilingual abstracts in the five official working languages of the United Nations. (PDF 406 kb) [file 40249_2019_541_MOESM1_ESM.pdf]

Translation of the abstract into the five official working languages of the United Nations

تعميم العوامل المرتبطة بعبء حمى الضنك في ولاية أوديشا في الهند خلال الفترة من عام 2010 إلى 2016

سبهاسيشا سوين (Subhashisa Swain) وميناكشي بهات (Minakshi Bhatt) وسانجاميترا باتي (Sanghamitra Pati) وريكاردو جي. سواريس ماجالهيس (Ricardo J. Soares Magalhaes)

الخُلاصة

تهدفُ هذه الدراسة لتقدير العبء الوبائي لحمى الضنك في ولاية أوديشا الهندية باستخدام الأساليب العلمية كمعدل السنة الحياتية للإعاقة (DALY) واستطلاع العوامل ذات الصلة في الأعوام ما بين 2010 إلى 2016. أثناء الفترة من عام 2010 إلى 2016، تم الإبلاغ عن 27772 حالة (68.4% من الذكور) في الولاية. كان متوسط العمر (بالسنوات) لكلٍ من الذكور والإناث هو 31.63 و 33.82 على التوالي. متوسط معدل السنة الحياتية للإعاقة لكل 100000 شخص على صعيد الولاية كان أعلى في عام 2016 (ما يعادل 0.45) ومتوسط عمر الأشخاص المفقودين فيما يخص معدل السنة الحياتية للإعاقة كان الأعلى في عام 2015 (ما يعادل 34.90 سنة). يشيرُ نموذج التراجع المعدّل أن الزيادة في كل وحدة بخصوص الرطوبة والكثافة السكانية تزيدُ معدل السنة الحياتية للإعاقة بمقدار 1.05 و 1.02 وحدة على التوالي. في حين أن تغيير الوحدة في نسبة نوع الجنس (الإناث لكل 1000 ذكر) والغطاء الحرجي تزيد معدل السنة الحياتية للإعاقة بمقدار 0.98 وحدة. تشيرُ نتائجنا إلى التباين الجغرافي لمعدل السنة الحياتية للإعاقة في ولاية أوديشا ذات الصلة بالكثافة السكانية والرطوبة والغطاء الحرجي. تقترحُ الاختلافات التي جرى تحديدها بين نسبة حدوث الحالات الاعتيادية والدليل التفصيلي لمعدل السنة الحياتية للإعاقة التي يمكنُ استخدامها لاحقًا لتوضيح عبء المرض بفعالية أكبر. يوحي معدل المرض الأكثر انتشارًا بين الذكور الشباب إلى الحاجة لتعزيز تدابير المكافحة والسيطرة.

Translated from English version into Arabic by Aalya Al-Beeshi, Revised by Nawal Sharabati, through

TRANSLATORS
WITHOUT BORDERS

2010–2016 年间印度奥里萨邦登革热的疾病负担分布及其相关因素

Subhashisa Swain, Minakshi Bhatt, Sanghamitra Pati and Ricardo J. Soares Magalhaes

摘要

本研究使用印度奥里萨邦登革热 2010–2016 年伤残调整寿命年（DALY）数据来估算其流行病学负担，并探讨相关因素。在 2010–2016 年间，该州共报告了 27 772 例（男性占 68.4%）登革热病例，男性和女性的平均年龄分别为 31.63 和 33.82（年）。每 10 万人平均伤残调整寿命年在 2016 年较高（0.45），人均 DALY 在 2015 年的损失最高（34.90 岁）。调整回归模型显示，每个单位的湿度和人口密度的增加分别导致 DALY 增加 1.05 和 1.02 个单位。然而，性别比例（每千名男性中的女性）和森林覆盖率的单位变化使 DALY 增加 0.98 个单位。研究结果表明，奥里萨邦 DALY 的地理分别与人口密度、湿度和森林覆盖率有关。 在标准发病率和 DALY 地图之间的差异表明，后者可更有效地呈现疾病负担。年轻男性中的流行率较高表明需要加强有针对性的预防和控制措施。

Translated from English version into Chinese by Qing-Yun Chen, edited by Jin Chen

CHINESE CENTER FOR DISEASE CONTROL AND PREVENTION
NATIONAL INSTITUTE OF PARASITIC DISEASES

Distribution du fardeau de la dengue et facteurs associés dans l'État indien de l'Odisha entre 2010 et 2016

Subhashisa Swain, Minakshi Bhatt, Sanghamitra Pati et Ricardo J. Soares Magalhaes

Résumé

Cette étude est destinée à estimer le fardeau épidémiologique de la dengue dans l'État de l'Odisha, en Inde, à l'aide de l'espérance de vie corrigée de l'incapacité (EVCI) et d'explorer les facteurs associés sur la période de 2010 à 2016. Au cours de la période 2010-2016, 27 772 cas de dengue (68,4 % d'hommes) ont été rapportés dans cet État. L'âge moyen était de 31,63 ans pour les hommes et 33,82 pour les femmes. L'espérance de vie corrigée de l'incapacité (EVCI) moyenne par 100 000 personnes dans les districts était plus grande en 2016 (0.45) et le nombre d'années d'espérance de vie perdues par personne était à son maximum en 2015 (34.90 années). Le modèle de régression ajusté indique que chaque unité d'augmentation de l'humidité et de la densité de population augmente l'EVCI de 1.05 et 1.02 unité, respectivement. En revanche, le changement d'unité sex-ratio (femmes pour 1000 hommes) et de la couverture forestière augmente l'EVCI de 0.98 unité. Nos résultats indiquent une variation géographique de l'EVCI dans l'Odisha qui est associée à la densité de population, à l'humidité et à la couverture forestière. Les différences identifiées entre l'incidence normalisée et les cartes d'EVCI suggèrent que ces dernières peuvent être utilisées pour prévenir plus efficacement la maladie. La prévalence plus élevée parmi les jeunes hommes suggère qu'il est nécessaire de renforcer les mesures de prévention et les contrôle ciblés.

Translated from English version into French by Suzanne Assenat, Revised by Emmanuelle Hardan, through

Распространенность денге и связанные с ней факторы в штате Одиша Индии в 2010-2016 гг.

Субхашиса Свейн, Минакши Бхат, Сангхамитра Пати и Рикардо Х. Соарес Магальяес

Аннотация

Настоящее исследование проводится для оценки эпидемиологической нагрузки от денге в штате Одиша в Индии на основе годов жизни, скорректированных по нетрудоспособности, и изучения связанных с ней факторов в 2010-2016 гг. В период с 2010 г. по 2016 г. в штате сообщалось о 27 772 случаях (68,4 % у мужчин). Доля заболевших мужского и женского пола составила, соответственно, 31,63 % и 33,82 %. Средняя продолжительность жизни,

скорректированная по нетрудоспособности, по всему региону в расчете на 100 000 человек была наивысшей в 2016 г. (0,45), а в расчете на человека наивысшей она была в 2015 г. (34,90 лет). Скорректированная регресионная модель показывает, что при повышении влажности и повышении плотности населения на единицу годы жизни, скорректированные по нетрудостпособности, возрастают, соответственно, на 1,05 и 1,02 единиц. При этом при изменении на единицу соотношения полов (число женщин на 1000 мужчин) и покрытия лесом годы жизни, скорректированные по нетрудоспособности, возрастают на 0,98 единиц. Наши результаты демонстрируют географическую вариабельность годов жизни, скорректированных по нетрудоспособности, в штате Одиша, связанную с плотностью населения, влажностью и покрытием лесом. Обнаруженные расхождения между стандартной встречаемостью и картой распростанения денге позднее можно использовать для получения более эффективного представления о бремени болезни. Преобладание болезни у молодых мужчин указывает на необходимость усиления целенаправленных мер профилактики и контроля.

Translated from English version into Russian by Alexander Somin, Revised by Michael Orlov, through

TRANSLATORS
WITHOUT BORDERS

Factores asociados a la infección por dengue y su distribución en el estado de Odisha, India, durante el periodo 2010-2016

Subhashisa Swain, Minakshi Bhatt, Sanghamitra Pati y Ricardo J. Soares Magalhaes

Resumen
Este estudio tiene como objetivo estimar el peso epidemiológico de dengue en Odisha, India, mediante los métodos DALY (años de vida ajustados por la discapacidad) y explorar los factores asociados durante los años 2010 a 2016. Durante el periodo de 2010 a 2016, se informó de 27 772 casos (68,4% masculinos) en el estado. La edad media de las personas, tanto hombres como mujeres fue de 31,63 y 33,82 años, respectivamente. El promedio de vida ajustado por discapacidad según el distrito (DALY) por 100 000 personas fue mayor en el año 2016 (0,45) y el promedio DALY perdido por persona fue el más alto en el año 2015 (34.90 años). El modelo de regresión ajustado indica que cada unidad de incremento en humedad y en densidad de población aumenta DALY en 1,05 y 1,02 unidades respectivamente. Mientras, el cambio de unidad en el cociente de sexo (femenino por 1000 masculinos) y la superficie forestal aumentan DALY en 0,98 unidades. Nuestros resultados señalan una variación de DALY en Odisha asociada a la densidad de población, la humedad y a la superficie forestal. Las discrepancias identificadas entre la incidencia estándar y los mapas DALY sugieren que este último puede usarse de forma más efectiva para presentar el peso de la enfermedad. La mayor prevalencia entre individuos jóvenes masculinos sugiere la necesidad de fortalecer las medidas de prevención y control específicas.

Translated from English version into Spanish by Ingrid Schulz, Revised by Patricia Cassoni, through

TRANSLATORS
WITHOUT BORDERS
